# Four novel nontuberculous mycobacteria species: Mycobacterium wendilense sp. nov., Mycobacterium burgundiense sp. nov., Mycobacterium kokjensenii sp. nov. and Mycobacterium holstebronense sp. nov. revived from a historical Danish strain collection

**DOI:** 10.1099/ijsem.0.006620

**Published:** 2025-01-07

**Authors:** Xenia Emilie Sinding Iversen, Erik Michael Rasmussen, Dorte Bek Folkvardsen, Erik Svensson, Conor J. Meehan, René Jørgensen, Anders Norman, Troels Lillebaek

**Affiliations:** 1 International Reference Laboratory of Mycobacteriology, Statens Serum Institut, Copenhagen, Denmark; 2 Department of Biosciences, Nottingham Trent University, Nottingham, UK; 3 Department of Science and Environment, University of Roskilde, 4000 Roskilde, Denmark; 4 Global Health Section, Department of Public Health, University of Copenhagen, Copenhagen, Denmark

**Keywords:** *Mycobacterium burgundiense *sp. nov., *Mycobacterium holstebronense *sp. nov., *Mycobacterium kokjensenii *sp. nov., *Mycobacterium wendilense *sp. nov., nontuberculous mycobacteria, novel species, taxonomic description

## Abstract

Four novel nontuberculous mycobacteria were discovered from a historical strain collection at the International Reference Laboratory of Mycobacteriology at Statens Serum Institut in Copenhagen, Denmark. Phylogenetic analysis combining the 16S *rrs*, internal transcribed spacer and 23S *rrl* elements, as well as a single-copy core-gene (*hsp65*, *rpoB+C*, *secA* and *tuf*) analysis of these freeze-dried mycobacteria, clinically isolated from gastric lavage samples between 1948 and 1955, showed to be associated with type strains grouping within the Terra and Fortuitum-Vaccae clade. Phenotypic characteristics, biochemical properties and fatty acid and mycolic acid profiles supported the classification as novel strains. A genomic comparison to the closest related type strain was done by calculating average nucleotide identity and *in silico* DNA:DNA hybridization values, which showed 87.9% and 33.0% for Mu0050, 85.2% and 27.4% for Mu0053, 85.3% and 27.6% for Mu0083 and 93.3% and 50.1% for Mu0102, respectively. The names proposed for the new species are *Mycobacterium wendilense* sp. nov. (Mu0050^T^=ITM 501390^T^=CCUG 77525^T^), *Mycobacterium burgundiense* sp. nov. (Mu0053^T^=ITM 501391^T^=CCUG 77526^T^), *Mycobacterium kokjensenii* sp. nov. (Mu0083^T^=ITM 501392^T^=CCUG 77527^T^) and *Mycobacterium holstebronense* sp. nov. (Mu0102^T^=ITM 501393^T^=CCUG 77528^T^).

## Introduction

The genus *Mycobacterium* was first described in 1896 by Lehmann and Neumann based primarily on phenotypic characteristics, and today, the genus contains just over 190 species with validly published names according to The International Code of Nomenclature of Prokaryotes [1]. This genus, which includes *Mycobacterium tuberculosis* and *Mycobacterium leprae* that cause severe human diseases, also encompasses a diverse group of so-called nontuberculous mycobacteria (NTM) [2]. While the genus was split into five genera in 2018, recent genomic analysis has re-combined these back into a single genus, *Mycobacterium* [3,4].

Human NTM disease has been associated with immunosuppression or structural lung damage. However, in recent years, NTM diseases have been documented in several immunocompetent individuals. Furthermore, many studies report an increasing global trend in NTM cases [5]. NTM found in various environmental niches are considered ubiquitous opportunistic pathogens that are contagious through the ingestion of contaminated water or food, inhalation of aerosols or inoculation [5,7].

Laboratory-based identification of mycobacteria has traditionally involved biochemical tests, colony morphology and pigmentation, together with optimal growth conditions and growth rate classification. The Runyon classification originally categorized species into two main groups: rapid-growing mycobacteria, where visible colonies form within 7 days, and slow-growing mycobacteria, whose colonies become visible after more than 7 days of incubation [8]. Later studies have revealed distinct groupings within these two classes, leading to the addition of an intermediate growth rate category. For example, *Mycobacterium heraklionense*, identified in 2013, is classified with an intermediate growth rate, with colonies appearing between 5 and 15 days of incubation [9]. The development of high-performance liquid chromatography (HPLC) and matrix-assisted laser desorption ionization-time of flight (MALDI-TOF) methods has later enabled identification with higher sensitivity, analysing species-specific lipid and mycolic acid profiles. Later implementation of 16S rRNA (Sanger) sequencing has been used for a more straightforward comparison of phylogenetic relationships between species [10]. A combination of increased access to diagnostics, better culture methods and advancements in DNA sequencing and routine laboratory techniques has led to a rise in microbiological isolation of NTM and the discovery of several novel NTM species [11,12]. Today, a combination of phenotypic and genotypic criteria, including growth rate, mycolic acid type, biochemical characteristics and whole-genome sequencing (WGS), are used to distinguish closely related species and identify novel ones [13].

Since 1948, the International Reference Laboratory of Mycobacteriology (IRLM) at Statens Serum Institut (SSI) in Copenhagen has maintained a collection of over 4000 mycobacterial isolates that have been lyophilized and frozen. This collection provides an extraordinary opportunity to gain new insights into the history and genetics of mycobacteria. A previous study of these historical strains revealed four novel NTM species candidates. These were clinically isolated from patients in Denmark from 1948 to 1955. The novel species differed from known NTM species, and their uniqueness was verified by WGS and subsequent core-gene phylogenetic analysis [14].

In the present study, we present these four novel *Mycobacterium* strains and provide their genotypic and phenotypic characteristics. The proposed names for the four strains, designated Mu0050, Mu0053, Mu0083 and Mu0102, are *Mycobacterium wendilense* sp. nov., *Mycobacterium burgundiense* sp. nov., *Mycobacterium kokjensenii* sp. nov. and *Mycobacterium holstebronense* sp. nov., respectively.

## Isolate selection, clinical data and culturing

In the recorded metadata, all four mycobacterial isolates included in this study were designated ‘saprophyte’. They were all isolated from gastric lavages and collected from female patients born between 1905 and 1940 who lived in different provinces in Denmark at the time of collection (Table 1). The records received from tuberculosis (TB) sanatoria or hospital departments provided no further details on patients’ medical histories.

**Table 1.** Sample and patient information on the four mycobacterial isolates included in this study

Isolate	Sample information			Patient information		
	Sample material	Sample received	Recorded designation	Year of birth	Sex	Province of residence
Mu0050	Gastric lavage	29 July 1948	Saprophyte	1905	F	North Jutland
Mu0053	Gastric lavage	18 March 1948	Saprophyte	1940	F	Bornholm
Mu0083	Gastric lavage	14 November 1949	Saprophyte	1911	F	Copenhagen City
Mu0102	Gastric lavage	3 June 1955	Saprophyte	1919	F	West Jutland

Each freeze-dried isolate was resuspended in Dubos medium and cultured for 2 weeks in mycobacteria growth indicator tubes (MGIT) containing Middlebrook 7H9 broth supplemented with 0.8 ml of Oleic Albumin Dextrose Catalase (OADC) at 37 °C using the BD BACTEC™ MGIT™ 960 Mycobacteria Culture System (BD Diagnostic Systems, Franklin Lakes, NJ, USA). Additionally, each isolate was grown on Löwenstein–Jensen (LJ) slant media (SSI Diagnostica, Hillerød, Denmark) at 35 °C. The live bacteria were handled in the biosafety level 3 laboratory at the IRLM. All four isolates grew successfully on both solid LJ slants and in liquid MGIT medium, and the presence of acid-fast rod-shaped bacilli was confirmed by Ziehl–Neelsen (ZN) and auramine–rhodamine staining, respectively.

## Species identification and WGS

The initial screening for NTM species was conducted using the GenoType Mycobacterium CM (v2.0) and GenoType Mycobacterium AS (v1.0) line probe assays (LPA; Hain Lifescience, Nehren, Germany). Each assay was prepared according to the published heat inactivation and cell-lysis protocol [15]. The genus *Mycobacterium* was confirmed for all four isolates, but no specific NTM species could be identified through the LPA approach (Table 2).

**Table 2.** Species identification results from Hain Genotype CM/AS LPA and whole-genome comparison using ANI and dDDH values, between isolates and species representatives from the genus *Mycobacterium*

Isolate	Genotype CM/AS LPA		Genome identity (% to closest match)			
	**Species**	CM/AS pattern	*Mycobacterium* type strain (DSM no.)	16S *rrs* gene	ANI	dDDH
Mu0050	*Mycobacterium* sp.	1–3/1–3	*M. chitae* (44633^T^)	98.7	87.9	33.0
Mu0053	*Mycobacterium* sp.	1–3/1–3	*M. chitae* (44633^T^)	98.0^*^	85.2	27.4
Mu0083	*Mycobacterium* sp.	1–3,10/1–3,12	*M. heraklionense* (46753^T^)	99.5^*^	85.4	28.1
Mu0102	*Mycobacterium* sp.	1–3,10/1–3,12	*M. virginiense* (100883^T^)	99.7^*^	93.3	50.1

*A better 16S rRNA blast hit was found for these isolates than the highest ANI-scoring isolate (Mu0053: *M. confluentis*=98.4%, Mu0083: *M. virginiense*=99.5%, Mu0102: *M. icosomasiliensis*=99.7%). See also Table S1.

To ensure sufficient amounts of DNA for WGS on the Oxford Nanopore Technologies (ONT) platform, each isolate was cultured in four MGIT tubes. The bacteria were collected, and DNA was extracted using the QIAamp® DNA Mini Kit, as previously described [14], with the following modification: after the ethanol precipitation step, 600 µl aliquots of supernatant from all four extractions were added to the same spin column, before continuing to the washing steps, to increase the amount of DNA per isolate in the final eluate. Sequencing libraries were prepared using the Native barcoding genomic DNA kit (with EXP-NBD104, EXP-NBD114 and SQK-LSK109; ONT, Oxford, UK) according to the manufacturer’s protocol (version NBE_9065_v109_revV_14Aug2019) using AMPure XP beads (Beckman Coulter) for the clean-up steps. Purified libraries were run on a single FLO-MIN106D flow cell, and raw ONT reads were generated using the guppy base-calling software (v3.6.0). Subsequently, NanoFilt (v2.0.0) was used to remove reads with an average quality score below Q7, and the remaining barcode and adapter fragments were removed using qcat (v1.1.0). *De novo* genome assembly was performed using the Flye assembler (v.2.7.1), with three read polishing iterations and an expected final genome size of 5 Mb. The resulting contigs were imported into Geneious (v9.1.8) and marked as circular if indicated in the Flye assembler summary file. Previously generated paired-end Illumina reads [14] were imported and used to polish ONT-assembled draft genomes by mapping reads for up to five iterations, using the medium-low sensitivity setting in the built-in Geneious mapping module and to circularize any remaining open genomes. Consensus sequences were generated using a threshold of 75% and a minimum coverage threshold of 10 reads. Lastly, remaining variants and ambiguous base calls were manually resolved through visual inspection of both ONT and Illumina’s reads mapped to the new hybrid consensus. Thus, each strain genome sequence was consistently verified throughout by both ONT and Illumina sequences. Since these were obtained from separated cultures, they represent independent verifications of the genome. Coverage for each sequencing technology has been included in Table 3. We checked genome completeness with CheckM (v1.2.2), which showed 99.5–100% completeness for all genomes.

**Table 3.** Genome properties of completed genome sequences of four novel isolates, next to their closest related type strains (see Table 2). Secondary values in the Mu0050 column are for its plasmid genome. C: circular genome topology, ONT: Oxford Nanopore Technologies

Property	Mu0050	Mu0053	*M. chitae*	Mu0083	*M. heraklionense*	Mu0102	*M. virginiense*
Genome size (bp)	5 224 726/23 059	5 453 374	5 461 769	4 341 397	5 061 737	4 794 273	4 980 142
G+C content (%)	68.6/64.6	67.7	69.0	68.6	68.0	67.5	67.5
Topology	C / C	C	C	C	C	C	C
No. plasmids	1	–	–	–	–	–	–
Illumina coverage (X)	115/385	92		86		77	
ONT coverage (X)	90/256	86		94		90	
Est. plasmid copy number	3						

Each genome assembly was compared to all species-level representative genome assemblies of the family *Mycobacteriaceae*, registered in the National Center for Biotechnology Information (NCBI) RefSeq database on 9 June 2022. The programs FastANI (v1.35; https://github.com/ParBLiSS/FastANI) and the genome-to-genome distance calculator (v3.0; https://ggdc.dsmz.de) were used to calculate average nucleotide identity (ANI) and *in silico*/digital DNA:DNA hybridization (dDDH) values, respectively [16]. Furthermore, full-length (1521–1535 bp) 16S rRNA sequences from each genome sequence were extracted with barrnap (v0.9; https://github.com/tseemann/barrnap) and used to search against the NCBI 16S rRNA database (bacterial and archaeal sequences) with the blastn tool [17]. Each 16S rRNA fragment was also later verified (with Sanger sequencing) upon depositing isolates to the Belgian Coordinated Collections of Microorganisms (ITM) culture collection. With three of the four isolates, we found matches with higher nucleotide identity than the corresponding best ANI match. Furthermore, with the isolates Mu0083 and Mu0102, we found a number of 16S rRNA matches with >98.65% nucleotide identity, a commonly used species-level threshold for the 16S rRNA gene [18]. We therefore calculated dDDH values for each match to confirm that they each had lower values than the highest ANI matching species (Table S1, available in the online Supplementary Material). We observed that all high-scoring 16S rRNA matches fell below both species-level thresholds of 95% ANI and 70% dDDH, respectively. We therefore remained confident that all four isolates represent hitherto unknown species within the genus *Mycobacterium* [19]. Results for the four highest ANI-scoring species and their closest determined type strain have been included in Table 2.

## Genome features

Complete genome annotation was performed using the NCBI Prokaryotic Genome Annotation Pipeline (PGAP; v5.2, build version: 2021-07-01.build5508; https://github.com/ncbi/pgap). We also used the PathogenFinder (v1.1) and ResFinder (v4.1) web services (http://www.genomicepidemiology.org/services) to assess pathogenicity and acquired antimicrobial resistance (AMR) from each genome sequence. All four isolates were predicted to be human pathogens by PathogenFinder due to the presence of pathogenic gene families, but no AMR genes were identified, indicating low intrinsic drug resistance. The estimated human pathogen probability scores (PPS) obtained for the proposed novel species revealed lower values for Mu0050 and Mu0053 (0.45 and 0.52, respectively) compared to Mu0083 and Mu0102 (0.66 and 0.85, respectively). The isolate with the highest PPS still exhibited a marginally lower score than known human pathogenic species of *Mycobacterium* such as *tuberculosis*, *avium* and *intracellulare*, which all scored over 0.95 when uploaded to PathogenFinder (data not shown). These results are summarized in Table 4.

**Table 4.** Annotation statistics of novel isolates, obtained using the NCBI PGAP, next to their closest related type strains (see Table 2). Values from type strains were obtained from NCBI RefSeq. PF: PathogenFinder, NA: Not available

Property	Mu0050	Mu0053	*M. chitae*	Mu0083	*M. heraklionense*	Mu0102	*M. virginiense*
CDS	4916	5127	5182	3932	4632	4376	4611
Pseudogenes	78	73	79	38	66	48	59
rRNA loci	2	2	2	2	2	2	2
tmRNA genes	1	1	1	1	1	1	1
tRNA genes	46	48	46	46	47	46	46
ncRNA genes	3	3	3	3	3	3	3
IS-elements	71	41	na	11	na	14	na
*rrs* Δ451–482*	Yes	Yes	Yes	No	No	No	No
Prophages	1	3	na	1	na	0	na
PF pathogen score	0.521	0.448	0.440	0.663	0.591	0.848	0.757

*Deletion in the 16S rRNA gene *rrs*, which is commonly found in rapidly growing mycobacteria [4,21].

Similar to the pathogen scores, the annotation statistics reveal a pairwise heterogeneity, with the largest correlation observed between Mu0050 and Mu0053, as well as Mu0083 and Mu0102, respectively. Similar observations were made with genome size and G+C content, but all observed values were consistent with members of *Mycobacterium*, with Mu0083 and Mu0102 showing similarities to the type strains belonging to the *Mycobacterium terrae* complex, which are characterized by relatively smaller genome sizes [20]. Additionally, a 23 Kbp circular plasmid with an estimated copy number of 3 was identified in the isolate Mu0050 (Table 3).

We also observed a similar pairing when looking at annotation statistics (number of coding sequences, pseudogenes, etc.). The 16S rRNA sequences of Mu0050 and Mu0053 both lacked the short 12–14 nucleotide insert in helix 18 between positions 451 and 482, whereas the 16S rRNA sequences of Mu0083 and Mu0102 contained the extended helix at the same position (Table 4). This deletion in *rrs* 451–482 is well-characterized and often used as a marker to distinguish between rapid- and slow-growing mycobacteria [4,21].

## Phylogeny

**Fig. 1.**
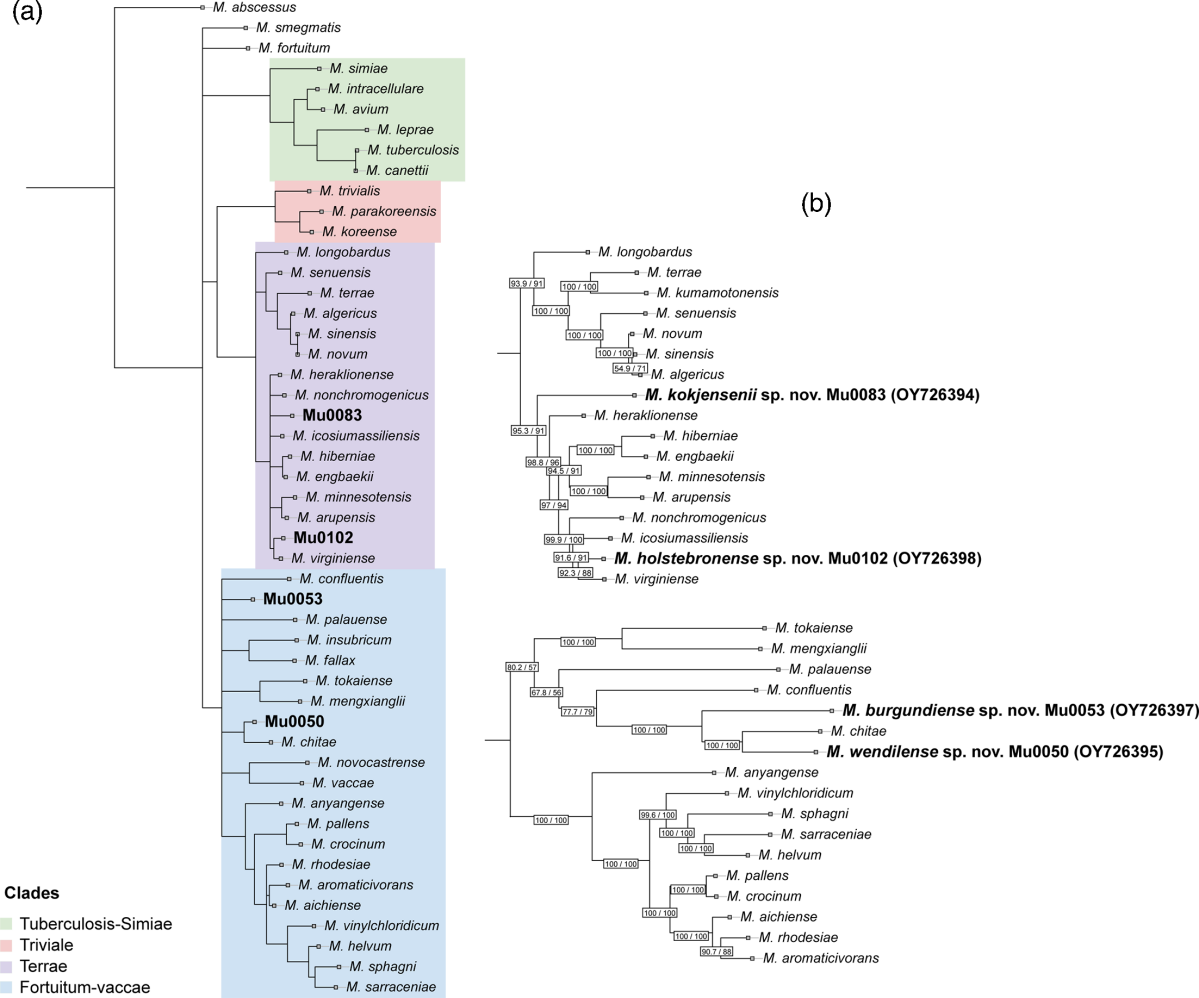
Maximum likelihood (ML) phylogenies of the four novel strains (Mu0050, Mu0053, Mu0083 and Mu0102), together with 44 selected reference genomes (80% ANI cutoff) generated using IQ-tree. The left tree (**a**) is a consensus tree of 1000 bootstrap replicates, based on the entire rDNA locus (16S *rrs*, ITS and 23S *rrl* concatenated). Clades are coloured according to their taxonomic grouping within the genus *Mycobacterium* [4]. The two trees on the right (**b**) show separate ML phylogenies generated using five concatenated single-copy core genes (*hsp65*, *rpoB +C*, *secA* and *tuf*) for the Terrae (top) and Fortuitum-Vaccae (bottom) clades. GenBank accessions for each strain genome are written in parenthesis next to their respective isolate along with their proposed new species names.

To determine the evolutionary placement of the four new isolates within the genus *Mycobacterium*, we generated a phylogeny using a subset of 44 reference genomes, which we selected based on an 80% ANI cutoff. This approach reduced computational complexity while adequately representing the most closely related species. Additionally, *Mycobacterium abscessus* was included to act as an outgroup. Due to the relatively poor discriminatory power of the 16S rRNA gene within the genus *Mycobacterium*, we elected to use the complete 16S–23S rDNA region, including the internal transcribed spacer (ITS) [22]. Sequences were first aligned using the software MAFFT, manually checked and curated for poorly aligned regions in Geneious. We then used IQ-tree to perform phylogenetic analysis [23]. The alignment was partitioned into 16S *rrs*, ITS and 23S *rrl* regions. ModelFinder was used to select the best-fit model of sequence evolution of each respective region according to the Bayesian information criterion. Ultrafast bootstrap analysis with 1000 replicates was conducted to generate a consensus tree with a 90% bootstrap support cutoff, which we visualized using the iTOL web service (Fig. 1a). From this consensus tree, we identified two robust clades, one containing the isolates Mu0050 and Mu0053 (corresponding to the ‘Fortuitum-Vaccae’ clade described by Gupta *et al*.) and one containing Mu0083 and Mu00102 (corresponding to the ‘Terrae’ clade) [4]. Each clade was subsequently analysed separately in the following manner for increased robustness: from each genome sequence, we extracted the five core genes *hsp65*, *rpoB*, *rpoC*, *secA* and *tuf*, each known to have relatively high discriminatory power within the genus *Mycobacterium*. We then employed a codon-based alignment approach, where sequences from each gene were translated into protein sequences, aligned using MAFFT and back-translated into their original DNA sequences to retain codon positions. The five alignments were then concatenated into one. We then used a partitioning model, where each gene was analysed separately, and first and second codon positions were evaluated separately from third codon positions to account for different rates of evolution. We then used IQ-tree with ModelFinder as described above, using ultrafast bootstrap and the SH-like approximate likelihood ratio test with 1000 replicates to calculate branch support values. The resulting two maximum likelihood phylogenies are shown in Fig. 1b. As expected, these phylogenies provided a more robust and detailed overview of species relation than the *rrs*–ITS*–rrl* rRNA region [24]. The two Terrae clade members were more closely related to surrounding type strains than the Fortuitum-Vaccae clade members (Mu0050 and Mu0053). However, the novel isolates (Mu0083 and Mu0102) showed clear separation with strong branch support, consistent with their novel species status based on below-threshold ANI and dDDH values, highlighting the limited discriminatory power of the 16S rRNA gene (see Tables 2 and S1).

## Physiology and chemotaxonomy

The four strains were characterized by a panel of phenotypic and biochemical tests using methods described by Murray *et al.* [25], with cultures isolated from MGIT and LJ. All tests were conducted in duplicate, and only young cultures were harvested and used for downstream tests and analyses. Growth rate and temperature tests were performed on Middlebrook 7H10 (MB7H10) agar plates (SSI Diagnostica, Denmark) at 22, 31, 35, 37 and 42 °C (Table S2), whereas pigmentation and colony morphology were studied using LJ media. Visible colonies for Mu0050, Mu0083 and Mu0102 appeared within 7 days, classifying these strains as rapid-growing. In contrast, Mu0053 displayed an intermediate growth rate, with visible colonies appearing after 10 days. All strains exhibited optimal growth at 35–37 °C, while none could grow at 42 °C. Additionally, a test on 5% blood agar (SSI Diagnostica, Denmark) was performed, and for all four strains, colonies appeared within 5 to 8 days. On LJ media, colonies of strains Mu0050 and Mu0053 showed small, smooth colonies measuring 1–2 mm in diameter with a cream-coloured/light brown pigmentation, similar to their closest related reference *M. chitae*. In contrast, strain Mu0083 and Mu0102 colonies were rough and had yellow pigmentation. The colonies of Mu0083 were 1 mm and noticeably paler than the 2 mm big Mu0102. Both isolates showed physiological differences compared to their closest relative, for which pigmentation is described as absent and the colony morphology for *M. heraklionense* is smooth. The panel of recommended biochemical reactions included the following: catalase activity, Tween 80 hydrolysis, growth on MacConkey agar, nitrate reduction and activity of pyrazinamidase and urease (Table 5). None of the strains was positive for heat-stable catalase activity; however, in the semiquantitative catalase test, Mu0050 differed by showing limited effervescence (<45 mm). None of the novel strains could grow on MacConkey agar without crystal violet in the inhibition test, but all strains were positive in the nitrate reduction assay, distinguishing Mu0050 and Mu0053 from *M. chitae*. Mu0050 and Mu0053 were distinct from Mu0083 and Mu0102 in their ability to hydrolyse Tween 80, demonstrating negative and positive activity, respectively. All isolates were cultured in BACTEC MGIT 960 PZA medium (pH=5.9) to test for pyrazinamidase activity and growth at lower pH values (BD Diagnostic Systems, Franklin Lakes, NJ, USA). A loss of pyrazinamidase activity was observed in all novel strains (positive culturing within 7–10 days) except Mu0050, which, similar to *M. chitae*, showed susceptibility to the antimicrobial drug pyrazinamide. A comparison with the closest related and previously described NTM species is also detailed in Table 5.

**Table 5.** Growth characteristics and biochemical tests of the four novel isolates compared to their closest related species. Data for related species were retrieved from the following literature; *M. chitae* [31], *M. heraklionense* [9], *M. virginiense* [32]. Growth rates: Rapid, <7 days; Intermediate, 5–12 days; Slow, >12 days

Property	Mu0050	Mu0053	*M. chitae*	Mu0083	*M. heraklionense*	Mu0102	*M. virginiense*
***Growth characteristics***							
Growth rate (days)	Rapid	Intermediate	Rapid	Rapid	Intermediate	Rapid	Slow
Optimal temperature (°C)	35–37	35	28–37	35	25–37	37	35
Growth at 42 °C	−	−	−	−	−	−	−
Pigmentation	Cream	Cream	Cream	Pale yellow	Absent	Yellow	Absent
Colony morphology	Smooth	Smooth	Smooth	Rough	Smooth	Rough	na
***Biochemical tests***							
Catalase (68 °C)	+	+	+	+	+	+	+
Semi-quantitative catalase test	< 45 mm	> 45 mm	na	> 45 mm	> 45 mm	> 45 mm	> 45 mm
Growth on MacConkey agar	−	−	−	−	−	−	na
Nitrate reduction	+	+	−	+	+	+	+
Pyrazinamide	+	−	+	−	na	−	+
Tween 80 hydrolysis	−	−	na	+	+	+	+
Urease	−	−	+	−	−	−	−
Growth on blood agar	+	+	+	+	+	+	na

## Chemotaxonomy

**Fig. 2.**
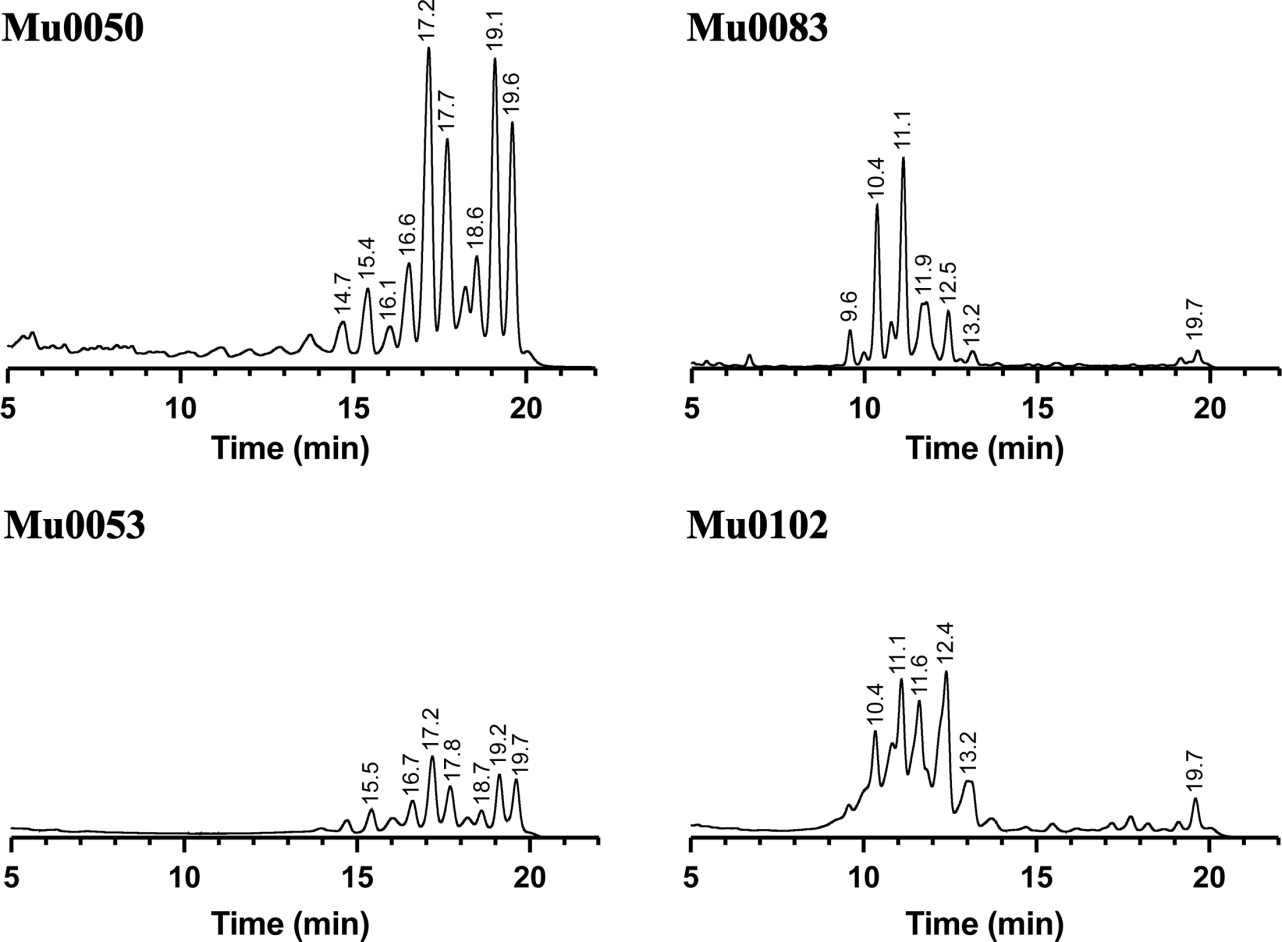
Mycolic acid HPLC patterns of the four novel strains.

HPLC was used to identify extracted mycolic acid patterns of the four novel strains using the protocol and instructions described by Hagen *et al.* and the Sherlock Mycobacteria Identification System (MIDI) [26]. Analyses were carried out on a Vanquish UHPLC system (Thermo Scientiﬁc) consisting of a quaternary pump (VF-P20-A), an autosampler with a thermostat and column compartment (VH-A10-A and VH-C10-A) and a ﬂuorescence detector (VF-D50-A). The ﬂuorometric detection wavelengths were set to 345 and 425 nm for emission and excitation, respectively. The data were acquired and processed using Chromeleon 7.2 SR4. Chromatographic separation was carried out on a Kromasil 100–5 C18 HPLC column with a particle size of 5 µm, dimensions 3.9×150 mm and used with a column temperature of 50 °C, an injection volume of 20 µl and an isocratic ﬂow of 1.0 ml min^−1^ for 23 min per sample. Each sample was eluded with a gradient from 10% to 90% of methanol and 2-propanol. The HPLC chromatograms were prepared using Prism 9 (GraphPad Software, Inc.) (Fig. 2). Mycolic acid patterns for Mu0050 and Mu0053 were very similar to the pattern of reference strain *M. chitae*, the closest related species within previous analyses, providing two significant double peaks within one cluster eluting between 15 and 20 min (Figs 2 and S1). HPLC of Mu0083 and Mu0102 proved to be closest to the mycobacteria of the *M. terrae* complex, displaying two separate peak clusters as related species, as described by Butler *et al.* [27]. For Mu0083 and Mu0102, the clusters peaked between 10 and 13 min and again at 19.7 min (Fig. 2).

**Fig. 3.**
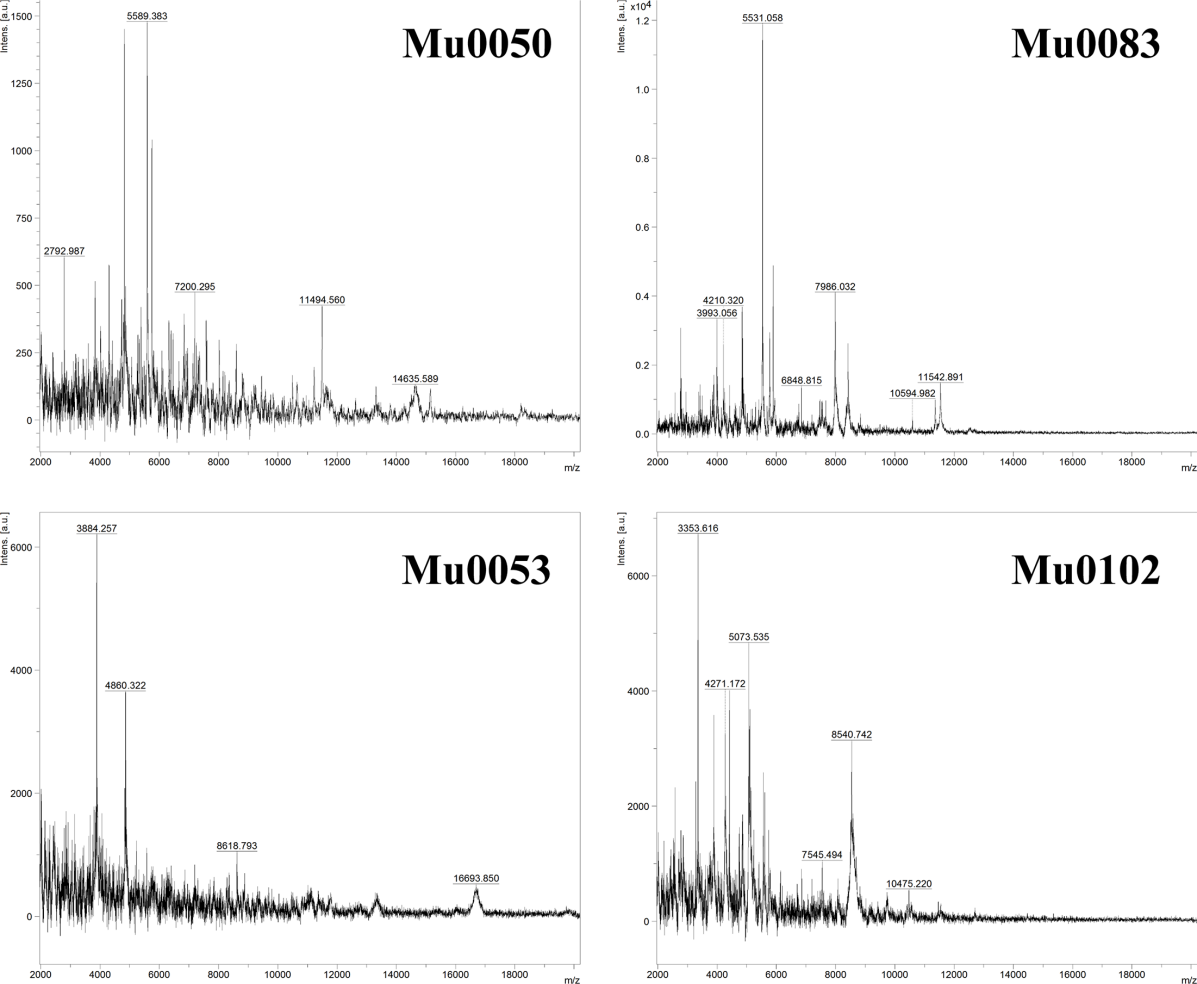
MALDI-TOF MS spectra of the four strains.

Duplicated MALDI-TOF mass spectrometry (MS) protein profiles for the four isolates were acquired using a Bruker Microflex LT/SH platform with the microbial identification software MALDI Biotyper® (MBT) Compass (v4.1). Cells were harvested from MGIT cultures, followed by inactivation and preparation according to the MALDI-TOF sonication protocol described by Alcaide *et al.* [28]. The MS profiles were obtained from technical duplicates and processed using Bruker Compass for the flexSeries 1.4 package, including flexAnalysis (v3.4) and flexControl (v3.4) software. MS spectra of the four strains did not match any spectral profiles of known mycobacteria included in the MBT Compass species identification programme. Nevertheless, they were assigned to the genus *Mycobacterium* and showed unique species-specific MS profiles (Fig. 3).

## Antimicrobial susceptibility testing

A phenotypic *in vitro* antimicrobial susceptibility testing (AST) of the four novel strains was performed using both Sensititre^®^ RAPMYCO and SLOMYCO plates and panel test kits (TREK Diagnostic Systems, Thermo Scientific, Cleveland, OH, USA) according to the manufacturer’s protocol to get a broad panel of drug susceptibilities. This broth-based microdilution method was designed based on Clinical and Laboratory Standards Institute guidelines, including relevant NTM drug options [29]. The two strains, Mu0083 and Mu0102, could not grow within the SLOMYCO plates with the kit-included cation-adjusted Mueller–Hinton broth (MHB) added to the OADC buffer. However, successful growth in the SLOMYCO plates was achieved by using cation-adjusted MHB with *N*-Tris(hydroxymethyl)methyl-2-aminoethanesulfonic acid (TES) buffer, which is provided for the RAPMYCO test kit. The minimal inhibitory concentrations (MICs) were read after 72 h of incubation at 37 °C. The testing for antimicrobial susceptibility also presented a pairwise concordance between the isolates Mu0050 and Mu0053, as well as Mu0083 and Mu0102, respectively (Table 6).

**Table 6.** AST showing MIC distributions of the novel strains Mu0050, Mu0053, Mu0083 and Mu0102 using TREK Sensititre® RAPMYCO and SLOMYCO plates with TES medium

Drug	Mu0050	Mu0053	Mu0083	Mu0102
SXT	1/19	4/76	>8/152	>8/152
CIP	≤0.12	≤0.12	4	1
MXF	≤0.12	≤0.12	4	1
FOX	64	64	>128	>128
AMI	≤1	≤1	4	4
DOX	0.5	0.5	16	8
TGC	0.25	1	>4	>4
CLA	64	>64	0.25	>64
LZD	4	2	4	2
IMI	64	>64	>64	>64
FEP	>32	>32	>32	>32
AUG2	64/32	>64/32	>64/32	>64/32
AXO	32	>64	16	16
MIN	≤1	≤1	>8	>8
TOB	4	2	≤1	≤1
RFB	0.5	4	≤0.25	≤0.25
EMB	4	4	>16	>16
INH	>8	>8	>8	>8
RIF	>8	>8	≤0.12	≤0.12
STR	2	1	64	32
ETH	>20	>20	>20	>20

## Discussion

This study introduces four new NTM species identified from the historical strain collection maintained at the IRLM at SSI, Copenhagen, since 1948. The unnamed species were isolated in the 1950s from pulmonary samples derived from patients, most likely under suspicion of TB. Unfortunately, due to a lack of detailed medical history, it remains unclear whether the identified NTM were the cause of lung disease, indicators of NTM colonization or simply transient findings. Nonetheless, given that pulmonary samples were procured from either TB sanatoria or hospital departments, we can presume the presence of TB-like symptoms in all four cases.

Our genomic analysis identified the three nearest known species to our novel strains: *M. chitae* for Mu0050 and Mu0053, * M. heraklionense* for Mu0083 and *M. virginiense* for Mu0102. These related mycobacteria fall into one out of four risk groups, based on their clinical relevance for humans and animals, according to European Union Directive 2000/54/EC. All three closest relatives to the novel species (*M. chitae*, *M. heraklionense* and *M. virginiense*) are assigned to group 2, which can cause illness but are unlikely to spread to the community and are usually treatable [30].

While categorizing these closely related species into risk group 2 matches their known low clinical significance, our PathogenFinder analysis predicts pathogenicity for each of the four novel species. Notably, Mu0083 and Mu0102 had higher scores when predicting pathogenicity than Mu0050 and Mu0053. Despite being isolated from patients presumably presenting with pulmonary TB-like symptoms, their clinical relevance remains uncertain. Therefore, thorough descriptions of these novel NTM are essential for future accurate species identification and for establishing robust epidemiological relationships.

Throughout our analyses, we noticed a trend of pairwise correlation between Mu0050 and Mu0053 and Mu0083 and Mu0102. However, despite these relationships, each of the four isolates represents distinct species, as their physiological and chemotaxonomic properties, mycolic acid and lipid profiles and phylogenetic placement attest. Therefore, these four historical mycobacterial strains each represent novel species of the genus *Mycobacterium*.

## Protologue

### Description of *Mycobacterium wendilense* sp. nov.

*Mycobacterium wendilense* (wen.di.len’se. M.L. neut. adj. *wendilense*, of Vendsyssel in the northern Jutland, Denmark, where the patient lived).

A non-motile, acid-fast, rod-shaped, Gram-positive and fast-growing bacterium. Growth on solid media requires culturing for <7 days at temperatures between 31 and 37 °C, optimally at 35 °C, producing small, smooth, cream-coloured colonies of 1–2 mm in diameter on Middlebrook 7H10 agar plates. This species produces <45 mm foam in the semiquantitative catalase test, is positive for heat-stable catalase and exhibits nitrate reduction. Reactions for urease activity and Tween 80 hydrolysis were negative, and no growth was attained when culturing at 42 °C nor on MacConkey agar without crystal violet.

The genome of type strain Mu0050^T^ (= ITM 501390^T^ = CCUG 77525^T^) comprises 5 224 726 bp *in silico*, and the G+C content is 68.6%. The whole-genome sequence and the 16S rRNA gene sequence are deposited in the NCBI GenBank database under the accession numbers GCA_963378085.1 and PP499327, respectively.

### Description of *Mycobacterium burgundiense* sp. nov.

*Mycobacterium burgundiense* (bur.gun.di.en’se. N.L. neut. adj. *burgundiense*, named after the island Bornholm – in Latin: Burgundia, where the patient lived in a small village).

The non-motile, acid-fast, rod-shaped and Gram-positive bacterium is growing at an intermediate rate. Growth on solid media requires culturing for >9 days at temperatures between 22 and 37 °C, optimally at 35 °C, producing small, smooth, light brown coloured colonies of 1–2 mm in diameter on Middlebrook 7H10 agar plates. This species produces >45 mm foam in the semiquantitative catalase test, is positive for heat-stable catalase and exhibits nitrate reduction. Reactions for urease activity and Tween 80 hydrolysis were negative, and no growth was attained when culturing at 42 °C nor on MacConkey agar without crystal violet.

The genome of type strain Mu0053^T^ (= ITM 501391^T^ = CCUG 77526^T^) comprises 5 453 374 bp *in silico*, and the G+C content is 67.7%. The whole-genome sequence and the 16S rRNA gene sequence are deposited in the NCBI GenBank database under accession numbers GCA_963378095 and PP499328, respectively.

### Description of *Mycobacterium kokjensenii* sp. nov.

*Mycobacterium kokjensenii* (kok.jen.se’ni.i. N.L. gen. n. *kokjensenii*, is named after Axel Kok-Jensen, a Danish pulmonologist who dedicated his life to combating lung diseases, particularly with a focus on mycobacteria and TB among socially disadvantaged population groups in Denmark).

A non-motile, acid-fast, rod-shaped, Gram-positive and fast-growing bacterium. Growth on solid media requires culturing for <7 days at temperatures between 22 and 37 °C, optimally at 35 °C, producing small, rough, pale yellow-coloured colonies of 1 mm in diameter on Middlebrook 7H10 agar plates. This species produces >45 mm foam in the semiquantitative catalase test, is positive for heat-stable catalase and exhibits nitrate reduction. Reactions for urease activity and Tween 80 hydrolysis were negative, and no growth was attained when culturing at 42 °C nor on MacConkey agar without crystal violet.

The genome of type strain Mu0083^T^ (= ITM 501392^T^ = CCUG 77527^T^) comprises 4 341 397 bp *in silico*, and the G+C content is 68.6%. The whole-genome sequence and the 16S rRNA gene sequence are deposited in the NCBI GenBank database under accession numbers GCA_963378075 and PP499329, respectively.

### Description of *Mycobacterium holstebronense* sp. nov.

*Mycobacterium holstebronense* (hol.ste.bro.nen’se. N.L. neut. adj. *holstebronense*, Holstebro, Denmark, referring to the place of isolation at the TB sanatorium in Holstebro, 1955).

A non-motile, acid-fast, rod-shaped, Gram-positive and fast-growing bacterium. Growth on solid media requires culturing for <7 days at temperatures between 22 and 37 °C, optimally at 37 °C, producing small, rough, yellow-coloured colonies of 2 mm in diameter on Middlebrook 7H10 agar plates. This species generates >45 mm of foam in the semiquantitative catalase test, tests positive for heat-stable catalase and demonstrates nitrate reduction and Tween 80 hydrolysis. Urease activity was negative, and the species failed to grow at 42 °C or on MacConkey agar without crystal violet.

The genome of type strain Mu0102^T^ (= ITM 501393^T^ = CCUG 77528^T^) comprises 4 794 273 bp *in silico*, and the G+C content is 67.5%. The whole-genome sequence and the 16S rRNA gene sequence are deposited in the NCBI GenBank database under accession numbers GCA_963378105 and PP499330, respectively.

## Repositories

**Table:** 

Isolate	Assembly id	Study id	Sample id	GenBank id	16S rRNA id
Mu0050	GCA_963378085.1	PRJEB50291	ERS16256601	OY726395	PP499327
Plasmid (pMu0050)				OY726396	
Mu0053	GCA_963378095	PRJEB50291	ERS16256603	OY726397	PP499328
Mu0083	GCA_963378075	PRJEB50291	ERS16256605	OY726394	PP499329
Mu0102	GCA_963378105	PRJEB50291	ERS16256607	OY726398	PP499330

## supplementary material

10.1099/ijsem.0.006620 Uncited Supplementary Material 1.

## Abbreviations

aLRT: approximate likelihood ratio test
AMR: antimicrobial resistance
ANI: average nucleotide identity
AST: antimicrobial susceptibility testing
BiC: Bayesian information criterion
CDS: protein-coding sequence
CLSI: Clinical and Laboratory Standards Institute
GGD: genome-to-genome distance
HPLC: high-performance liquid chromatography
IRLM: International Reference Laboratory of Mycobacteriology
ITS: internal transcribed spacer
LJ: Löwenstein–Jensen
LPA: line probe assay
MALDI-TOF: matrix-assisted laser desorption ionization-time of flight
MB7H10: Middlebrook 7H10
MBT: MALDI Biotyper®
MGIT: mycobacteria growth indicator tube
MHB: Mueller–Hinton broth
MIC: minimal inhibitory concentrations
MS: mass spectrometry
NCBI: National Center for Biotechnology Information
NTM: nontuberculous mycobacteria
ONT: Oxford Nanopore Technologies
PGAP: Prokaryotic Genome Annotation Pipeline
PPS: pathogen probability scores
SSI: Statens Serum Institut
TB: tuberculosis
TES: *N*-Tris(hydroxymethyl)methyl-2-aminoethanesulfonic acid
WGS: whole-genome sequencing
ZN: Ziehl–Neelsen
