# Supplementary material for: Four novel nontuberculous mycobacteria species: Mycobacterium wendilense sp. nov., Mycobacterium burgundiense sp. nov., Mycobacterium kokjensenii sp. nov. and Mycobacterium holstebronense sp. nov. revived from a historical Danish strain collection
Source: Int J Syst Evol Microbiol. 2025 Jan 7;75(1):006620. doi: 10.1099/ijsem.0.006620 (PMC11706282; doi:10.1099/ijsem.0.006620)

Supplementary Material

Supplementary Table 1. Comparison of identity scores (percent) of 16S rRNA (blastn hits) to type strain sequences and whole genome average nucleotide identities (ANI) and digital DNA:DNA hybridisation (dDDH) values for all species where 16S rRNA identities were above 98.65% (a commonly used species-level threshold), or higher than the higest ANI-scoring species. The higest scoring species is marked in bold for each category.

Species name	16S	ANI	dDDH
**Mu0050**			
M. chitae	**98.70**	**87.90**	**33.0**
**Mu0053**			
M. confluentis	**98.40**	81.14	21.6
M. chitae	98.00	**85.17**	**27.4**
**Mu0083**			
M. virginiensis	**99.54**	85.30	27.6
M. heraklionensis	99.51	**85.40**	**28.1**
M. arupensis	99.46	84.10	26.2
M. icosiumassiliensis	99.41	84.80	27.8
M. paraterrae	99.22	79.34	20.5
M. engbaekii	99.12	84.47	26.4
M. nonchromogenicus	98.98	84.76	26.9
M. hiberniae	98.86	84.35	26.4
M. minnesotensis	98.72	83.80	26.6
**Mu0102**			
M. icosiumassiliensis	**99.74**	90.60	41.7
M. virginiensis	99.67	**93.30**	**50.1**
M. arupensis	99.53	86.10	29.8
M. nonchromogenicus	99.49	89.00	35.9
M. engbaekii	99.32	86.30	29.8
M. paraterrae	99.29	78.80	19.6
M. minnesotensis	99.19	86.14	29.7
M. hiberniae	99.07	86.20	29.7
M. heraklionensis	99.02	90.40	39.9

Supplementary Table 2. Growth (colony count; semi-qunatitative scale) on Middlebrook 7H10 agar plates of the four novel isolates at five different temperatures.

Isolate	22°C	31°C	35°C	37°C	42°C
Mu0050	-	+	++	++	-
Mu0053	+	++	+++	++	-
Mu0083	+	++	+++	++	-
Mu0102	+	++	++	+++	-

Supplementary Figure 1. HPLC chromatogram of type culture strain *M. chitae* (CCUG 39504^T).

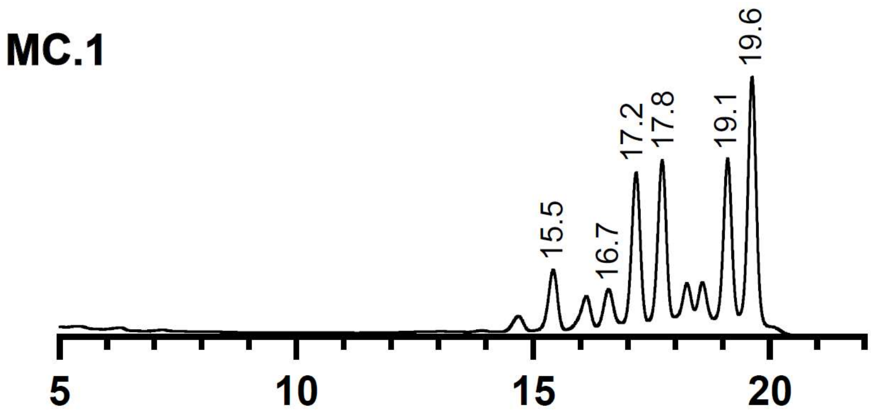

Supplement: Uncited Supplementary Material 1. [file ijsem-75-06620-s001.pdf]
